# Security Analysis and Improvements of Authentication and Access Control in the Internet of Things

**DOI:** 10.3390/s140814786

**Published:** 2014-08-13

**Authors:** Bruce Ndibanje, Hoon-Jae Lee, Sang-Gon Lee

**Affiliations:** 1 Department of Ubiquitous IT Graduate School of Design & IT, Dongseo University, Sasang-Gu, Busan 617-716, Korea; E-Mail: ndibanje.bruce.phd@ieee.org; 2 Division of Computer & Engineering, Dongseo University, Sasang-Gu, Busan 617-716, Korea; E-Mail: nok60@dongseo.ac.kr

**Keywords:** Internet of Things, wireless sensor networks, mutual authentication, access control

## Abstract

Internet of Things is a ubiquitous concept where physical objects are connected over the internet and are provided with unique identifiers to enable their self-identification to other devices and the ability to continuously generate data and transmit it over a network. Hence, the security of the network, data and sensor devices is a paramount concern in the IoT network as it grows very fast in terms of exchanged data and interconnected sensor nodes. This paper analyses the authentication and access control method using in the Internet of Things presented by Jing *et al* (Authentication and Access Control in the Internet of Things. In Proceedings of the 2012 32nd International Conference on Distributed Computing Systems Workshops, Macau, China, 18–21 June 2012, pp. 588–592). According to our analysis, Jing *et al.*'s protocol is costly in the message exchange and the security assessment is not strong enough for such a protocol. Therefore, we propose improvements to the protocol to fill the discovered weakness gaps. The protocol enhancements facilitate many services to the users such as user anonymity, mutual authentication, and secure session key establishment. Finally, the performance and security analysis show that the improved protocol possesses many advantages against popular attacks, and achieves better efficiency at low communication cost.

## 1. Introduction

Today, there is a multitude of envisioned and implemented use cases using smart devices and sensing nodes thus forming an emerging global and Internet-based information service platform called the Internet of Things (IoT) [1]. According to the ITU concept, the fundamental IoT design can be perceived like practically each physical thing around the world would be able precisely, “things” are not transformed to become computers, but they have tiny computers' abilities in a tiny foorprint and smarter nature [2]. IoT involves many technologies, including architecture, sensor/identification, coding, transmission, data processing, network, discovery, *etc.*

**Figure 1.**
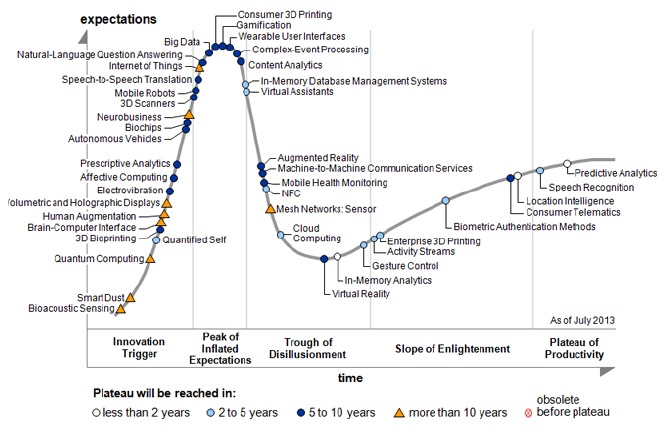
Gartner 2013 Hype Cycle of emerging technologies.

Kevin Ashton, cofounder and executive director of the Auto-ID Center at MIT, was the first to coin the term Internet of Things in 1999 in the context of supply chain management [3]. Nevertheless, in the past decade, this concept has been extended because of new IoT network applications such as e-healthcare and transport utilities [4]. The evolution of the IoT has its origin in the convergence of wireless technologies, advancements of microelectromechanical systems (MEMS) and digital electronics where has been as a result miniature devices with the ability to sense and compute and communicate wirelessly. In the era of IoT, the interaction or relationship between humans and machines is ever more considered as machines getting smarter and starting to handle more human tasks, and in this situation humans are required to trust the machine and feel safe. In this way, a *thing* might be a patient with a medical implant to facilitate real-time monitoring in a healthcare application or an accelerometer for movement attached to the cow in a farm environment. Figure 1 depicts the Hype Cycle for Emerging Technologies report which is the longest-running annual Hype Cycle [5].

The most challenging topics in such an interconnected system of miniaturized “things” are security and privacy aspects [6–10]. Authentication and access control technologies [11–19] are known as the central elements to address security and privacy problems in computer networks [20–33]. They can prevent unauthorized users from gaining access to resources, prevent legitimate users from accessing resources in an unauthorized manner, and enable legitimate users to access resources in an authorized manner. When building an IoT infrastructure, it is paramount to take in consideration the efficiency, security scalability and market oriented computing, power resource and storage features for the best quality of services to provide the costumers or users.

In 2012, Jing *et al.* proposed an authentication and access control method using the IoT [20]. Their paper mainly analyzes existing authentication and access control methods; also they design a feasible protocol for the Internet of Things. According to their scheme, in the authentication protocol they focused on simple and efficient secure key establishment based on ECC. For the access control policy, they adopted the Role Based Access Control (RBAC)-based authorization method using the thing's particular role(s) and application(s) in the associated IoT network. In this paper, we show that their scheme is costly in the whole communication process for the sensor nodes in the IoT, and also the security assessment they proposed is not practical in a working scenario. After an obvious analysis we propose improvements to their protocol in terms of security and computation cost and finally a comparative performance analysis with existing schemes is done to evaluate our proposal. The main contributions of this paper are security improvements at a reasonable computation cost. In order to make the scheme work solidly and to meet the security services requirements in the IoT we first format the Jing *et al.* protocol by separating their protocol into the main knows steps of protocol standards such as registration phase (offline or online), login and verification phase. In addition, we incorporate an important function named recovery or change password allowing users to modify their passwords in case of need. Therefore, every user will need to register with the HRA server during the registration phase. The purpose of this phase is to negotiate and compute different secret parameters for the login and authentication process between the user and a gateway node. The mutual authentication process is a combination of login and verification phases. Secondly we contribute in term of terms of performance analysis by analyzing the computation cost using different metric parameters such as: time to perform one way hash computation (TH), cryptosystem (RC5, ECC,…), random number generation function (R) in comparison with related works and finally we provided a security analysis in regard of known network and data attacks.

The rest of the paper is structured as follows: Section 2 presents the related works in the IoT field with security as main key point. Section 3 reviews the Jing scheme and performs a detailed cryptanalysis of that protocol, while Section 4 suggests improvements to the Jing scheme. The security analysis of the enhanced scheme is done in Section 5, before concluding this paper in Section 6.

## 2. Related Works

The IoT field is rapidly gaining attention given its capability of information collection and transmission by connecting everything through the internet. A certain number of researches projects are being carried out at different universities and labs to achieve the best quality of service in the area. The security aspect is among the research topics under study and more solutions have been proposed. In this section we present a review on the works done in this area.

Jingjun and Liangmin [34] presented a rapid identification authentication protocol for mobile nodes which is a convenient kind of protocol in the environment of the Internet of Things with privacy protection where the mobiles nodes are required to be authenticated by the cluster in order to perform the communication. The protocol designed is based on the Veronoi [35] network model and it contains a valid request message and an answer authentication message, which rapidly implements identification authentication and privacy protection. Moreover the authors analyzed the protocol security and finally they formalized the protocol in applied pi calculus which is a language for describing concurrent processes and their interactions. It extends the pi calculus adding the possibility to model cryptographic primitives through a signature and an equational theory. This is to prove the privacy protection properties in the protocol. In comparison with existing single-step protocols like the basic hash protocol and OSK protocol, the authors found that their protocol has less communication overhead, is secure enough and presents more privacy protection aspects compared to the related protocols.

Liang *et al.* [36] proposed security-critical multimedia service architecture in the IoT context for multimedia applications with important characteristics such as traffic analysis, security requirements and traffic scheduling. According to the authors, their proposal is one of the first security-aware traffic management strategies for such applications in the IoT. The major components of the proposed protocol are as following: key management [37–39], batch rekeying, authentication and watermarking. The proposed scheme in the authentication process involves methods ranging from the use of access control and capability certificates to mutual authentication between the server and user based on the access control, ability certificates and mutual authentication [40,41]. Generally, the function of watermarking is about indentifying the content origin, to trace illegally distributed materials and prevent unauthorized content access [42]. To accommodate different multimedia application needs, three modes of operation are suggested [43]: periodic batch rekeying, periodic batch leave rekeying and periodic batch join rekeying.

Gao *et al.* [44] suggested a communication protocol for RFID systems in the Internet of Things and proved its safety by the random oracle method [45]. The proposed security model for RFID systems in the IoT mainly consists of readers, tags and RFID middleware. Each object in the system has a unique EPC. In order to describe the RFID system model in the Internet of Things the random oracle model is applied [46]. The article proposes the SPAP protocol which uses symmetric encryption, one-way hash function and XOR. As proved by the random oracle model, SPAP can achieve mutual authentications, internal security, ownership transfer of tags; what's more, SPAP can also resist retransmission, tracking of some basic attacks. Finally, according to the safe performance analysis results, the SPAP protocol has good performance.

More recently Ye *et al.* [47] have proposed an efficient authentication and access control scheme for the perception layer of the Internet of Things focused on simple and efficient mutual authentication and secure key establishment based on ECC, which has much lower storage and communication overheads. The ABC-based authorization method has been adopted for the access control policy. Their architecture design is mainly based on the concept of a base station (BS) which collects the data and controls the sensor nodes, the user is defined as a visitor in the perception layer, including devices such mobiles phones, and smart computers. Finally the attribute authority (AA) is the entity in charge of creating and managing the attribute information. An efficient ECC-based authentication and the attribute-based access control policy were proposed in order to achieve mutual authentication between user and nodes and fine-grained access control. Mutual authentication ensures the security of the communication between user and nodes, whose process is simple to solve the resource-constrained problem of the IoT perception layer. Accessing the data on the basis of user attribute certificates in the access control authority can achieve flexible fine-grained access control. The proposed scheme has better performance on the sensor node side in comparison with others reported in [48].

## 3. Review of Jing *et al.*'s Method and Cryptanalysis

### 3.1. Overview of Jing et al.' Scheme

**Figure 2.**
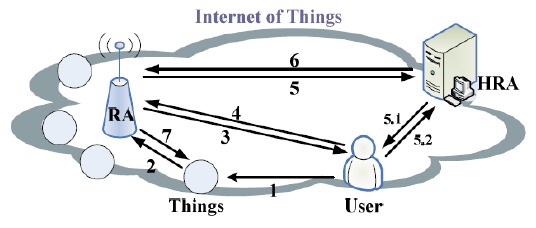
IoT architecture example.

This section assesses the work done by Jing *et al.* in its whole communication process. First, based on ECC, the authors propose an authentication protocol for an efficient secure key establishment. Second, after addressing some problems raised by the proposed protocol, a novel scheme for user access control in IoT has been adopted: the RBAC model. Figure 2 describes an architecture example of IoT given by the authors.

As shown in Figure 2, a complete request procedure for accessing a “Thing” involves seven steps:

*Step 1*: User requests to access a “Thing”;
*Step 2*: “Thing” sends an authentication request to its RA for verification purposes;
*Step 3*: RA requests User ID;
*Step 4*: User responds with HRA information;
*Step 5*: RA verifies the user's HRA information and sends an ID verification request to the HRA;
*Step 5.1*: HRA challenges the user with a question;
*Step 5.2*: User responds to the challenge with an answer;
*Step 6*: HRA responds if ID is OK or not;
*Step 7*: RA responds to the “Thing” about the user ID and issues a session key for the user.

The purpose of the authentication protocol is to provide access to the IoT to legitimate users. The authors suggested the use of the home registration authority (HRA) where all users are registered. According to the authors, “Things” or objects become end nodes in the Internet environment. They have unique global addresses (e.g., IPv6 address) and are capable of communicating with each other over the Internet. The exchanged messages for the proposed protocol are described in Figure 2 where exchanged messages between all involved entities (*User, Things, RA and HRA*) follow the aforementioned seven steps. Only an authenticated entity among the IoT can access the pervasive network to get the service requested. The *RA* verifies the certificate contents and the identity of the “*Thing*” and reviews the contents in order to determine if the information accurately describes the user. We summarize in Table 1 the notations used throughout this paper and their corresponding definitions.

#### 3.1.1. Review of the Authentication Protocol

The key establishment and distribution are the fundamental tasks for the entity authentication process. Based on the ECC algorithm, the authors believe it to be a solid solution to be considered. Therefore, to establish a session key in a given communication manner between two entities (taking as an example a user and object), the authors proposed three steps as follows:

- Step I: the *RA* who is responsible for the object will produce a random *P*∈*G* and compute *Ps* = *sP* in *Fp*. Note that s is a secret key that is assumed to be assigned before the *RA* has joined the IoT. For each user with *IDu*, *RA* will generate *Pu* = *h* (*IDu*) and the private key of the thing *Su* = *s Pu*.
- Step II: the user generates an ephemeral private key *a* and computes *Qu* = *a Su* and *Qu*′ = *a P.* Then the user will send an authentication message {*IDu*, *Qu*, *h* (*IDu*‖*IDt*‖*Qu*‖*Qu*′)} to the *RA*. Once the message is received, *RA* will compute *Qu*″ = *s*^−^*^1^Qu* and check whether *h* (*IDu*‖*IDt*‖*Qu*‖*Qu*″) equal to *h* (*IDu*‖*IDt*‖*Qu*‖*Qu*′) or not. If not, authentication fails. Otherwise go to step III.
- Step III: the session key establishment. Similarly, the *RA* will choose a random ephemeral key *b* and compute *Qt* = *bP* for the desired “*Thing*”. The session key will be *h* (*abP*) based on the ECC algorithm.

According to the authors, the next question is how to authenticate a legitimate user in the IoT. “Things” and users are in different domains. They could be located in different hierarchy levels of the network. The idea in [12] has been adopted to support their protocol design. As such, user authentication is performed in the user domain or a registered OpenID service provider. The authors denote it as home registration authority (*HRA*). Note that, peer-to-peer authentication method is another solution that can be utilized for further research. However, without solving the mutual-trust problem between two entities, this approach cannot succeed.

#### 3.1.2. Review of the Access Control Method

In the scheme proposed by the authors, they raised the problems of high computation load and more memory usage by the *RA*. To come up with solutions in the IoT, the authors argued that a novel scheme for user access control in the IoT would provide solutions for the problems addressed above. In this case, if a communication quality is already ensured, the access control algorithm decides whether a new connection is accepted. When applying role-based access control in the IoT network, the data and resources are only available to the users with access rights. It also supports three well known security principles: information hiding, least privilege, and separation of duties.

### 3.2. Cryptanalysis of Jing's Method

This subsection describes some weakness discovered in the scheme proposed by Jing *et al.* First of all, their scheme lacks clear details about the whole authentication process regarding the exchanged messages. Moreover, they did not separate the main known steps of a normal authentication process such as *registration phase* (*offline or online*) and *login phase*. Also, the contribution to the access control aspect lacks a scheme proposal.

#### 3.2.1. Session Key Establishment

**Figure 3.**
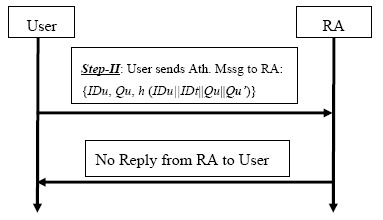
Unilateral authentication message.

When reviewing how the key session computation and establishment are done in the Jing *et al.*'s protocol, we found the following problems:

*Problem I:* In the second step of the session key computation and establishment, after computing the required parameters, the user sends an authentication message to the *RA*. Unfortunately, to meet the mutual authentication security service requirements, the *RA* after checking the received message, does not send a reply message to the user. In this analysis we found that their protocol is vulnerable to compromised device attacks and replay attacks, especially in Step 2. Figure 3 presents the no-mutual authentication protocol as aforementioned.

#### 3.2.2. Excess of Message Exchanges

The whole exchanged messages in the order to access the things raises some questions when analyzing the complete request procedure message (the seven steps), below is the problem found:

*Problem II:* The authors assumed that, among other roles, the *RA* has the role of pre-registration of the user *“every object will pre-register on a nearby trustworthy access point or gateway (denoted as Registration Authority, or RA)”*.

The HRA also has the role to register the users before network deployment. Following this analysis, there are unfortunately a mismatch in Step 3 where the RA sends a user ID request to the user and it is supposed to be pre-registered with the RA. Furthermore, in step 5.1: a challenge is sent to the user but, it is stated that all users are registered before network deployment in the HRA. In view of the fact that the author's protocol work over the IoT and the “Thing” defines the end node which does not require big storage capacity and are powerless, this analysis reveals that the step 3, step 4, step 5.1 and step 5.2 are excessive, hence causing high energy consumption and the need for high memory usage of the user device.

#### 3.2.3. Role Based Access Control

The authors propose the utilization of the access control instead of the ECC algorithm for the key session computation and authentication phases:

*Problem III:* Jing *et al.*'s protocol can solve the issues of high power consumption and memory storage of the RA by using the access control method. Unfortunately, this paper lacks any description of the RBAC method to support their theoretical explanation about how RBAC could work in this protocol if it came to replace the traditional methods. Thus we found that there is a need of a RBAC method proposal to strength their research article. However, the RBAC is out of the scope of our research area, so we don't touch this subject.

## 4. Proposed Improvements

The proposed improvements consist of two phases—registration phase and authentication phase—and one additional important function named password recovery or change. For convenience, the updated Table 2 below provides a new list of some notations and symbols to be used throughout the rest of paper, others symbol will be explained whenever they are used.

After analyzing the proposed scheme by Jing *et al.* in the IoT, this subsection presents the proposed enhancements. To fill this security gap, we propose security patches, which overcome the weakness found in the scheme of Jing *et al.* Before any detailed discussion of the proposed improvements, some assumptions are made and are supposed not to be violated while executing the scheme. The assumptions are mentioned below.

1. In the IoT, all the clients (*user, things, RA*) and service providers are supposed to be honest in the registration phase.
2. After the registration phase is over, no client (*user, things, RA*) and Server (*HRA*) is trusted. The clients need to verify themselves during login phase by providing exact identification data to access services and applications.
3. Once mutual authentication is performed, the *HRA* is always trusted and it is assumed that the server never compromises with the network adversaries.
4. To save the energy of the sensor nodes in the IoT, the user will only communicate with the gateway (*RA*) which acts as a sink and performs the mutual authentication.
5. *S* is a secret key that is assumed to be assigned before the *RA* has joined the IoT (Table 1).

### 4.1. Registration Phase

**Figure 4.**
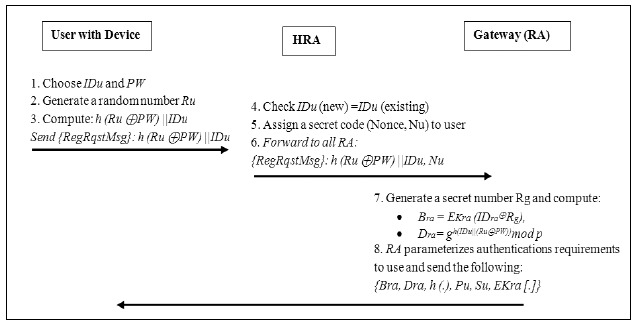
Registration phase flow.

In the registration phase, initially, each user must register with the *HRA* server. The aim of this phase is to allow users and a gateway node to negotiate a shared secret key for login and authentication success. As already mentioned in Jing *et al.*'s scheme for each user with *IDu*, RA will generate *Pu* = *h* (*IDu*) and the private key of the thing *Su* = *s Pu*. To process the registration phase, the following steps are required of the involved entities as given in Figure 4:

1. With his *IDu* the user chooses the *PW*,
2. Generates a random number *Ru* and computes *h (Ru*⊕*PW)*‖*IDu*.
3. The user submits the message to the *HRA* for a registration request to the *RA*.
4. *HRA* checks *IDu* (new) = *IDu* (existing). If equal, then he rejects the registration request otherwise,
5. Assign a Nonce *Nu* to the user and proceed to the next step.
6. The HRA forwards *h (Ru*⊕*PW)*‖*IDu* and *Nu* to *RA*.
7. Upon receiving the message from *HRA*, the *RA* generates a secret number *Rg* and computes the following:
  - *Bra* = *EKra (IDra*‖*Rg)*,
  - *Dra* = *g^(IDu^*‖*^(Ru^*‖*^PW))^mod p*
8. Afterward, the *RA* personalizes the authentication required parameters of the user with the *{Bra, Dra, h (.), Pu, Su, EKra [.]}*. The *RA* sends the reply message with the above parameters through the *HRA* which forward the message to the user. Here, *h* (.) is a collision free one-way function, e.g., SHA-1. The user now enters *Ru* into the smart card and it contains *{Bra, Dra, h (.), Pu, Su, EKra [.]}* The *RA* store the *IDu* in the table of ids to maintain it for login and authentication steps, this is the end of the registration phase.

### 4.2. Authentication Phase

**Figure 5.**
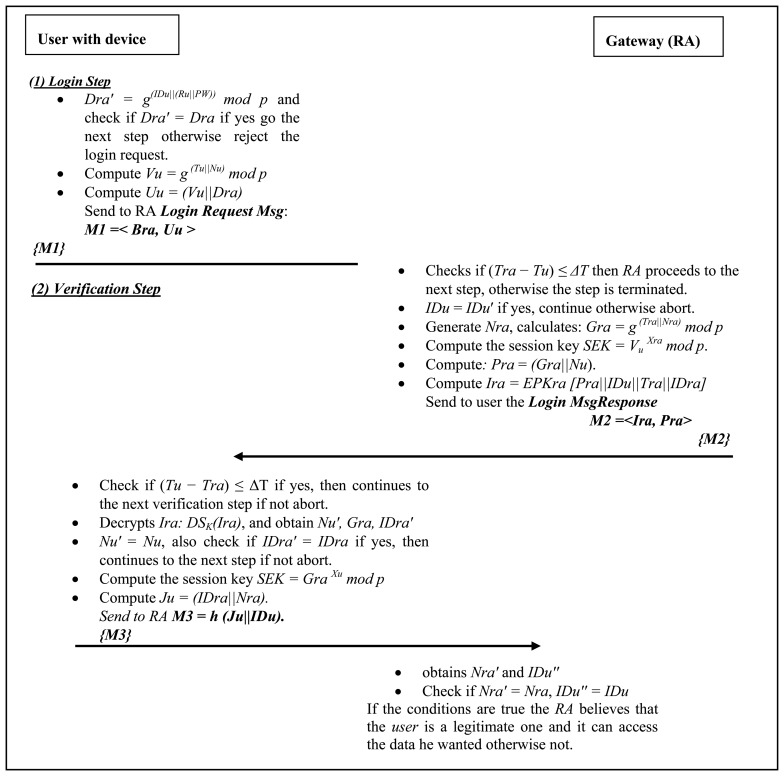
Authentication phase: login and verification steps flow.

This subsection describes the authentication phase as shown in Figure 5. It is divided into two steps as follows:

(A) *Login Phase:* This phase is invoked when the *user* wants to access the IoT network. In this proposed improvement protocol, the *user* doesn't communicate with the *thing* as it was structured in the original proposal. From our analysis, it is obvious that this step costs a lot in terms of energy because the *things* have to authenticate the *user* at every login demand. The computation of the mutual authentication consumes a lot of energy of the *things* this is why we limit the mutual authentication phase to the *RA*.
  After that, the *user* logs in to his device and inputs his *IDu* and *PW*. The local system of the smart device performs the following operations:
  Step 1-LP: Compute *Dra*′ = *g*^*(IDu*‖*(Ru*‖*PW))*^ *mod p* and check if *Dra*′ = *Dra* if yes go the next step otherwise reject the login request.
  Step 2-LP: Compute *Vu* = *g*^*(Tu*‖*Nu)*^ *mod p*. Here *Tu* and *Nu* are respectively the timestamp and nonce of the user device. Compute *Uu* = *(Vu*‖*Dra)*
  Step 3-LP: The user sends the login request message *M1* =< *Bra, Uu* > the *RA.* This is the end of the login step from the user to the *RA*, the message is sent over a public channel.
(B) *Verification Phase:* The verification phase is performed in order to mutually authenticate the user by the RA and *vice versa* while he wants to access the data from the IoT. Upon receiving the login request *M1* =< *Bra, Uu* > at time *Tra*, the *RA* authenticates *user* by the following steps:
  Step 1-VP: Checks if (*Tra* − *Tu*) ≤ *ΔT* then *RA* proceeds to the next step, otherwise the step is terminated. Here ΔT shows the expected time interval for the transmission delay and *Tra* is the time stamp of the gateway node.
  Step 2-VP: From the IDs table of the *RA* verify if *IDu* = *IDu*′ if yes, then the gateway considers that this is a legitimate user and proceeds to the next step, otherwise, the operations are terminated.
  Step 3-VP: The *RA* generates a nonce *Nra* then calculates *Gra* with the following: *Gra* = *g*^(*Tra*‖*Nra*)^ *mod p* and *RA* computes the session key *SEK* = *V_u_^Xra^mod p*. Here *Xra* is the secret number of the registration authority. Subsequently the *RA* computes *Ira* = *EPKra [Pra*‖*IDu*‖*Tra*‖*IDra]]* and sends to the user the message *M2* =<*Ira, Pra*> to respond to the login message request in order to process the mutual authentication. Here *Pra* = *(Gra*‖*Nu)*.
  After receiving the message M2 from the *RA*, the *user* perform the mutual authentication operations as follows:
  Step 4-VP: The user validates the time *Tra* and checks if (*Tu* − *Tra*) ≤ *ΔT* if yes, then continue to the next verification step and if not abort.
  Step 5-VP: From message M2, the user decrypts the message *Ira*, *DS_K_(Ira)* and checks if *Nu*′ = *Nu*, and also checks if *IDra*′ = *IDra.* If yes, then continues to the next step if not abort. The user calculates the session key with the knowledge of *Gra* from the decryption of *Ira*:
    $$\text{SEK}={\text{Gra}}^{Xu}\text{mod}\;p.$$
  Step 6-VP: After checking every parameter, the *user* can trust that the *RA* is the authentic one, and then *user* sends the last message M3, to acknowledge the session key from the Registration Authority:
    $$\begin{array}{l} M3=h\left(Ju∥\text{IDu}\right).\\ \text{Here}\;Ju=(\text{IDra}∥\text{Nra}) \end{array}$$
  After receiving the message M3, the Registration Authority performs the following steps:
  Step 7-VP: The RA computes the session key and decrypts the sub-message, obtains *Nra*′ and *IDu*″. The RA checks if *Nra*′ = *Nra*, *IDu*″ = *IDu*, if the conditions are true the *RA* believes that the *user* is a legitimate one and it can access the data he wanted, otherwise not.
  Step 8-VP: Furthermore, user and the *RA* share the session key *S_EK_* to perform subsequent operations during a session and the establishment of the session key terminates the authentication phase.

### 4.3. Password Change Procedure

In this subsection, we introduce the password-change/update phase. In the password-change phase, when a user wants to change his password *PW* to a new password *PW_Fresh_*, the following actions are taken into consideration:

Step-PCP1: The user performs a login operation as he did when he logged into the IoT by entering his *IDu* and password *PW*.
Step-PCP2: Initially, the local system of the user device validates the *user*'s entered *IDu* and *PW* with the stored values and if they match, the local system computes:
  $${\text{Dra}}^{\prime }=g^{\left(\text{IDu}\|Ru\|PW\right)}\text{mod}\;p$$
Step-PCP3: The user checks if *Dra*′ = *Dra*, if not, then the password change request is terminated; otherwise, proceed to the next steps.
Step-PCP4: Now, the user input his new password into the device which computes the operations with the user's fresh password:
  $${\text{Dra}}_{\text{new}}=g^{\left(\text{IDu}\left\|Ru\right\|PW_{\text{Fresh}}\right)}\text{mod}\;p.$$
Step-PCP5: The user's device replaces *Dra* by *Dra_new_*. Now, the new password is successfully changed and this phase is terminated.

## 5. Performance and Security Analysis

In this section, we present our proposed protocol evaluation in terms of security analysis, in [49–52] it was shown that the security services are taken into consideration more when analyzing the data and network security, so in this analysis we assume that an adversary may intercept M1, M2, and M3 at anytime. Also, we assume that an adversary may hack either passwords or *steal* a user device, *extract secrets*, but cannot do both at the same time. As per the current literature, extracting secrets from a smart card's memory is quite difficult and some smart card manufacturer companies provide countermeasures against the risk of such side channel attacks. Based on the above assumptions, an attacker may execute certain attacks to breach the proposed protocol.

### 5.1. Security Analysis

*Identity management*: The *RA* stores all the registered *ids* in the id management table and checks the availability of a unique id in each new registration phase. Furthermore, the ids are kept and transmitted over the IoT network in an encrypted form. In this case, the improved protocol is secure against node privacy threats.

*Mutual authentication*: Our proposed enhanced protocol provides mutual authentication, in the messages *M2* =<*Ira, Pra*> and *M3* = *h (Ju*‖*IDu)*, the user device and *RA* achieve the mutual authentication messages and both them can be sure that they are the legitimate ones.

*Confidentiality*: In particular, these messages are confidential from any attacker. As in most cases the communication in the IoT network is done over the open air where uncountable messages float and this might be an attractive situation for attackers. From this analysis, we suppose that an attacker can easily capture sensitive information while the messages are being transferred. The proposed protocol provides adequate confidentiality to the messages (such as *EPKra [Pra*‖*IDu*‖*Tra*‖*IDra]*, and *h(Ju*‖*IDu*). Hence, an attacker cannot extract any valuable information from the open air messages.

*Resist replay attacks***:** Our proposed protocol is resistant to replay attacks, because the authenticities of messages *M1*, *M2*, are timestamped and nonce-based. They are validated by checking the freshness of timestamps (((*Tra* − *Tu*) ≤ *ΔT*, (*Tu* − *Tra*) ≤ *ΔT*) and nonce (*Nu*′ = *Nu*, *Nra*′ = *Nra*). Suppose that an attacker intercepts a login request message *M1* and attempts to access the IoT by replaying the same message (*M1*). The verification of this login attempt fails, since the time difference expires (*i.e.*, (*Tra* − *Tu*) ≥ *ΔT*). In the same way, if he intercepts *M2 or M3* and tries to extract < *Ira*, *Pra, Ju* > and attempts to replay one of them, the verification request will fail because the time difference will expire again and also, the nonce will show that the message was already used. Hence, our protocol is secure against replaying of messages.

*Man-in-the-middle attacks*: An attacker may attempt a man-in-the-middle (*MIMT*) attack by modifying the login message *M1* =< *Cra, Uu* > to M1* =< Cra*, Uu* >. Nevertheless, this malicious attempt will not work, as the false IDu* will not be verified at the *RA* and the *RA* cannot get the original sub-message *(Vu*‖Dra)* by computing Uu*. Thus, man-in-the-middle attacks are not applicable to our protocol.

*Offline-password guessing attacks*: The password and id guessing attacks are not feasible for our proposed system because it lacks a verifier table. The login phase, passwords and ids are not transmitted in plain text; instead, they are hashed and some operations are performed with them. They are transmitted with some other secret (*i.e.*, *Dra* = *g*^*(IDu*‖*(Ru*‖*PW))*^ *mod p*), which makes it difficult for users to guess them.

*Securely change/update password*: The proposed protocol help users change passwords at any time if they forget it or if they get hacked this password change facility provides robustness to the proposed improved protocol in comparison with a static password-based protocol.

*Session key establishment*: This scheme provides session key establishment after the authentication phase. A session key [*i.e.*, *SEK* = *Gra^Xu^mod p*] is set up between the used device and the RA for secure subsequent communications. For each login session, the session key will be different and cannot be replayed after the time expires. Furthermore, the *user* and *RA* can securely execute encryptions and decryptions by using of the session key and hence, achieve confidentiality for the subsequent messages.

### 5.2. Performance Evaluation

The performance evaluation of the proposed improvements is based on the computation and communication costs in comparison with existing or related work [20,44,47,48]. The metrics used in this performance evaluation are listed below:

TH: Time to perform one way hash computation
S: Cryptosystem (*RC5, ECC, EK/DK,Private/Public/Session or Shared Key computation*)
R: Random number generation function
MUL: Executing ECC point multiplication
ADD: XOR operation

**Figure 6.**
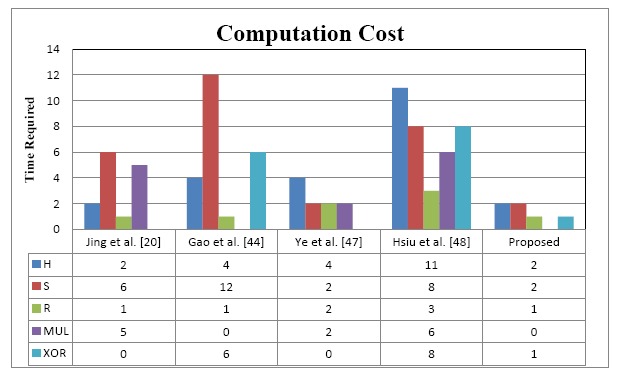
Authentication phase: login and verification steps flow.

The performance analysis gives the output of a statistical estimation of the computational cost, and communication cost from the comparison performance Figure 6, where the proposed improved protocol in term of computation cost, requires 2TH and 2 symmetric cryptosystems whereas in [20], [44], [47] and [48] 2TH+6S, 4TH+12S, 4TH+4S and 11TH+8S are required, respectively, in their complete protocols. Regarding other parameters, 1R, 1R, 2R and 3R are needed to perform the random number generation in [20], [44], [47] and [48] while1R is needed in our scheme. For the MUL parameter the proposed scheme does not use this operation and [44] neither. But 5, and 2, 6 times are needed for MUL in [20], [47] and [48]. In case of XOR operation 1 time in our scheme is needed, 6 and 8 times are required in [44] and [48] respectively while [20] and [47] don't use it.

**Figure 7.**
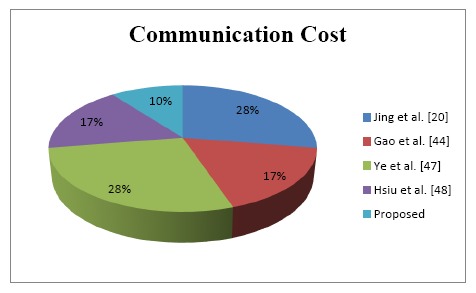
Authentication phase: login and verification steps flow.

As described in Figure 7, the cost of the communication in the improved protocol is lower than in other schemes due to the fact that our protocol architecture does not allow the user to interact directly with the “*things*” nodes. In terms of *thing* energy cost, only the user will access the data from the gateway (*RA*) which saves the energy of the *thing*, which is why we have more computation than other schemes when the users' devices are interacting with the *RA*.

In addition, we have separated the steps into different phases (registration phases and authentication phase). Thus, *thing* nodes consume less energy than other protocols. The performance analysis of the communication cost indicates that, the proposed improvements require three messages to fulfill all the communication and authentication process among the IoT devices. Figures 6 and 7 illustrate the aforementioned metrics in term of computation and communication cost. The proposed protocol achieves better efficiency at low communication cost because it requires only 10% (three exchanged messages compared with existing work) to finish the whole protocol process.

## 6. Conclusions

In this work, we have analyzed and improved Jing *et al.*'s protocol for the IoT. First we reviewed their work and analyzed it in details by a cryptanalysis methodology in order to find the problems in the proposed protocol and we found that their protocol is vulnerable to compromised device attacks and replay attacks. Second, we provided enhancements for different aspects corresponding to the security gaps found in their protocol. Furthermore, we have performed an evaluation of the proposed enhancements by security and performance analysis in term of computation and communication cost using selected metrics in comparison with recent research in the IoT area. Finally, the results of both security and performance analyses reveal that the improved protocol satisfies the demands of the key security services in the IoT such as confidentiality, integrality and authenticity and achieves better efficiency at a lower communication cost.

## Figures and Tables

**Table 1.** Notations and description.

**Notations**	**Descriptions**
*F_p_*	Finite field
*E*	Elliptic curve defined on *F_p_* with large order
*P*	Point on *E*
*G*	Group of elliptic curve points on *E*
*H (.)*	One-way hash function
*S*	*RA*'s private key
*IDu*	Identity of user
*IDt*	Identity of the “thing”
*RA*	Registration authority
*HRA*	Home registration authority
*IoT*	Internet of Thing
*ECC*	Elliptic curve cryptosystem
*RBAC*	Role based access control

**Table 2.** Updated table.

**Symbol**	**Description**
*PW*	Password of *IDu*
*Nu*	Generated Nonce by HRA to User
*MAC*	Unique Identity number of the device
*Nra*	Generated Nonce for the gateway
*IDra*	User ID of the gateway
*EK[m]*	Message *m* is encrypted with symmetric *key*
*DK[m]*	Message *m* is decrypted with symmetric *key*
*⊕*	Bitwise XOR operation
‖	Concatenation operation
